# Cytokine Release Syndrome in Patients Treated With Chimeric Antigen Receptor T-cell Therapy: A Retrospective Study Analyzing Risks, Outcomes, and Healthcare Burden

**DOI:** 10.7759/cureus.49452

**Published:** 2023-11-26

**Authors:** Rushin Patel, Mrunal Patel, Fehmida Laxmidhar, Khushboo Lakhatariya, Darshil Patel, Zalak Patel, Safia Shaikh

**Affiliations:** 1 Internal Medicine, Community Hospital of San Bernardino, San Bernardino, USA; 2 Internal Medicine, Trumbull Regional Medical Center, Niles, USA; 3 Clinical Research, Rush University Medical Center, Chicago, USA; 4 Internal Medicine, University of California Riverside School of Medicine, Riverside, USA; 5 Internal Medicine, Washington University School of Medicine, St. Louis, USA

**Keywords:** healthcare burden, outcomes, crs, cytokine release syndrome, car t, chimeric antigen receptor t-cell

## Abstract

Background

Chimeric antigen receptor T-cell (CAR-T) therapy has emerged as a promising immunotherapy for various malignancies. However, its use is associated with challenges, including cytokine release syndrome (CRS), a potentially severe complication. This retrospective study aims to analyze the risks, outcomes, and healthcare burden of CRS in patients undergoing CAR-T therapy.

Method

Data from the 2020 National Inpatient Sample (NIS) were utilized, comprising 415 CAR-T-related hospitalizations. They were categorized into those with CRS (n = 68) and those without CRS (n = 347). Baseline characteristics, including age, gender, race, income, insurance status, and comorbidities, were compared. Outcomes of interest included in-hospital mortality, length of stay (LOS), total hospital charges, and access to complications, associations, and interventions. Statistical analyses, including multivariable models, were employed to assess associations.

Results

Hospitalizations with CRS did not exhibit significant differences in age, gender, race, income, or insurance status compared to those without CRS. The multivariable analysis showed no statistically significant difference in mortality (adjusted odds ratio (aOR) = 2.48, 95% confidence interval (CI): 0.71 to 8.69, p = 0.151), LOS (coefficient = -2.1 days, 95% CI: -5.43 to 1.21, p = 0.207), or total hospital charges (coefficient = $207,456, 95% CI: $6119 to $421,031, p = 0.057) between the two groups. The CRS group had a higher incidence of fever (aOR = 1.91, 95% CI: 1.15 to 3.17, p = 0.014), acute respiratory failure (aOR = 2.10, 95% CI: 1.01 to 4.40, p= 0.049), and the need for intubation/mechanical ventilation (aOR = 2.59, 95% CI: 1.14 to 5.88, p = 0.024). Hemophagocytic lymphohistiocytosis (HLH) was significantly associated with CRS (aOR = 6.72, 95% CI: 2.03 to 22.18, p = 0.002).

Conclusion

While the development of CRS in CAR-T-treated patients did not significantly increase mortality, LOS, or total hospital charges, it was associated with specific risks and outcomes, including fever, respiratory failure, and HLH. This study emphasizes the importance of vigilance in recognizing and managing CRS in CAR-T therapy to optimize patient outcomes. The findings contribute valuable insights to guide clinical decision-making in the context of CAR-T therapy.

## Introduction

Chimeric antigen receptor T-cell (CAR-T) therapy is a form of immunotherapy in which a patient's T cells are collected and genetically modified to improve their targeting ability and facilitate the elimination of cancer cells [1]. CAR-T cells have been effective in treating various types of B-cell lymphomas, leukemias, and multiple myeloma, resulting in long-lasting remissions and often eliminating the cancer cells completely [2-5].

Even though CAR-T therapies are promising, they are not without their challenges. Most patients relapse and experience significant treatment-related toxicity, including cytokine release syndrome (CRS) and immune effector cell-associated neurotoxicity syndrome (ICANS), which have substantial health and economic implications [6].

CRS is a systemic inflammatory response that can be triggered by a variety of factors such as infections and certain drugs [7]. The primary manifestation of CRS is a fever, alongside a range of symptoms, including fatigue, muscle aches, gastrointestinal problems (nausea, vomiting, diarrhea), tachycardia, and skin rashes [8]. CRS can also result in life-threatening complications, including cardiac dysfunction, adult respiratory distress syndrome, neurotoxicity, renal failure, disseminated intravascular coagulation, multiorgan failure, and circulatory collapse [9]. Elevated levels of various cytokines, especially interleukin-6 (IL-6) and interleukin-2 (IL-2), are found in the serum of patients experiencing toxicities due to CRS, such as after CAR-T-cell infusions. The immune system is continuously interacting with cytokines released by CAR-T cells or macrophages, which creates a complex interplay [10]. CRS usually develops after the first week of CAR-T cell infusion, and with proper management, symptoms often subside within one to two weeks [11]. It is possible to predict CRS severity based on clinical factors such as disease burden, marrow involvement, lymphodepletion, and high CAR-T doses, as well as patient-specific factors such as pre-existing inflammation (baseline serum ferritin) and baseline endothelial activation (thrombocytopenia) [12]. Due to the potential occurrence of serious adverse events associated with CAR-T therapy, the FDA has implemented a Risk Evaluation and Mitigation Strategy (REMS). This mandates only skilled personnel to be able to administer this therapy and necessitates continuous availability of drugs like tocilizumab in case of adverse events. Tocilizumab, an IL-6 receptor antagonist, manages severe CRS from CAR-T cells without harming T cells, while blinatumomab, a bispecific antibody that redirects effector T cells to B cells with its anti-CD3 and anti-CD19 arms, aims to prevent CRS through strategies such as disease reduction, corticosteroids, and dose adjustment [13].

Despite the available treatment strategies for CRS, CRS associated with CAR-T therapy still carries the risk of morbidity and mortality. Therefore, we conducted a retrospective study to analyze the risks, outcomes, and healthcare burden of CRS in patients who underwent CAR-T therapy.

## Materials and methods

This retrospective study utilized data extracted from the National Inpatient Sample (NIS), a database supported by the Agency for Healthcare Research and Quality (AHRQ) and part of the Health Care Cost and Utilization Project (HCUP) [14]. The NIS database represents a roughly 20% stratified sample of discharges from almost 1000 US hospitals across all 50 states. It is important to note that the NIS database is the largest publicly available database in the United States for inpatient care, covering all types of payers. For this analysis, we worked with the 2020 NIS database, which includes hospitalizations occurring from January 1, 2020, to December 31, 2020. Additionally, because the NIS data is de-identified, this study was exempt from the need for Institutional Review Board (IRB) approval.

Study population

We employed the International Classification of Diseases, Tenth Revision, Clinical Modification (ICD-10-CM) codes to identify hospitalizations related to CAR-T therapy. Subsequently, we divided these hospitalizations into two distinct groups: one with CRS and the other without CRS. Specifically, for CAR-T hospitalizations, we used the ICD-10 codes XW033C3 and XW043C3, while for CRS cases, we utilized the ICD-10 codes D89.83, D89.831, D89.832, D89.833, D89.834, D89.835, and D89.839. We excluded categories such as "No charge," "Other," and "Missing value" for the primary payer (insurance status). Our inclusion criteria were restricted to adults aged 18 years and older.

Outcomes of interest

Our primary areas of interest included in-hospital mortality, length of stay, and the overall costs during the hospital stay. Our secondary goals involved exploring complications and associations related to CRS. To assess mortality, we used the NIS variable "DIED," and the length of hospital stays was determined through the NIS variable "LOS." To calculate the total charges associated with hospitalization, we used the variable "TOTCHG." Additionally, to access complications, associations, and interventions, we used the pertinent ICD-10 codes as provided in the Supplemental Table.

Statistical analysis

All analyses were conducted in accordance with the Healthcare Cost and Utilization Project regulations, which involved appropriate stratification, clustering, and weighting of samples [15]. We calculated odds ratios for binary variables and coefficients for continuous variables. Initially, we performed univariate analyses to determine unadjusted odds ratios. Subsequently, in the multivariable analysis, we included only those variables that showed a significant association with the outcome of interest in the univariate analysis, with a significance level of P < 0.05. To construct a multivariate analysis model, we incorporated potential confounding variables, such as age, gender, race, income quartile based on zip code, hospital division, hospital bed size, insurance status, and the Charlson Comorbidity Index score. The Charlson Comorbidity Index score encompasses conditions like myocardial infarction, congestive heart failure, peripheral arterial disease, cerebrovascular disease, dementia, chronic obstructive pulmonary disease, connective tissue disease, peptic ulcer disease, liver disease, diabetes, hemiplegia or paraplegia, chronic kidney disease, diabetes with end-organ damage, solid tumors, leukemia, lymphoma, and AIDS/HIV, all of which are conditions associated with high mortality rates [16].

Categorical variables were compared using the Fisher exact test, and continuous variables were compared using the Student's t-test. All P-values were two-sided, and the threshold for statistical significance was set at P < 0.05. The statistical analysis was carried out using STATA version 17, developed by StataCorp LLC in College Station, TX.

## Results

We identified 415 inpatient encounters linked to CAR-T therapy. Among them, 68 had CRS, while 347 did not. Table 1 presents the baseline characteristics of CAR-T hospitalizations, categorized into those with CRS and those without CRS, and provides details on characteristics such as median age, sex, race, median household income, insurance status, Charlson Comorbidity Index score, admission type, census division, and hospital bed-size, along with corresponding p-values.

**Table 1:** Baseline characteristics of CAR-T therapy hospitalizations CRS: cytokine release syndrome; CAR-T: chimeric antigen receptor T-cell.

	With CRS	Without CRS	Total CAR-T therapy hospitalizations	P-value
Number of hospitalizations	68	347	415	
Mean age	59.1 years	60.3 years	60.1 years	P = 0.517
Sex				P = 0.506
Male	43 (63.24%)	208 (59.65%)	251 (60.24%)	
Female	25 (36.76%)	139 (40.35%)	164 (39.76%)	
Race				P = 0.795
White	53 (78.46%)	268 (77.22%)	321 (77.42%)	
Black	6 (9.23%)	26 (7.4%)	32 (7.69%)	
Hispanic	5 (6.15%)	33 (9.47%)	38 (8.93%)	
Asian or Pacific Islander	3 (4.62%)	10 (2.96%)	13 (3.23%)	
Native American	0 (0%)	1 (0.3%)	1 (0.25%)	
Other	1 (1.54%)	9 (2.66%)	10 (2.48%)	
Median household income				P = 0.766
0-25th percentile	10 (14.93%)	58 (16.82%)	68 (16.5%)	
26th to 50th percentile	15 (22.39%)	68 (19.52%)	83 (20.0%)	
51st to 75th percentile	15 (22.39%	94 (27.03%)	109 (26.25%	
76th to 100th percentile	28 (40.30%)	127 (36.64%)	155 (37.25%)	
Insurance status				P = 0.533
Medicare	24 (34.85%)	153 (44.01%)	177 (42.50%)	
Medicaid	5 (7.58%)	25 (7.19%)	30 (7.25%)	
Private insurance	38 (56.06%)	164 (47.31%)	202 (48.75%)	
No insurance	1 (1.52%)	5 (1.50%)	6 (1.50%)	
Charlson Comorbidity Index score				P = 0.038
0	1 (1.47%)	0 (0%)	1 (0.24%)	
1	0 (0%)	1 (0.29%)	1 (0.24%)	
2	44 (64.71%)	191 (55.04%)	235 (56.63%)	
3 or more	23 (33.82%)	155 (44.67%)	178 (42.89%)	
Admission type				P = 0.445
Non-elective	21 (30.88%)	92 (26.51%)	113 (27.23%)	
Elective	47 (69.12%)	255 (73.49%)	302 (72.77%)	
Census division				P = 0.215
New England	6 (8.82%)	35 (10.09%)	41 (9.88%)	
Middle Atlantic	8 (11.76%)	64 (18.44%)	72 (17.35%)	
East North Central	12 (17.65%)	57 (16.43%)	69 (16.63%)	
West North Central	7 (10.29%)	29 (8.36%)	36 (8.67%)	
South Atlantic	11 (16.18)	54 (15.56%)	65 (15.66%)	
East South Central	2 (2.94%)	6 (1.73%)	8 (1.93%)	
West South Central	2 (2.94%)	32 (9.22%)	34 (8.19%)	
Mountain	2 (2.94%)	9 (2.59%)	11 (2.65%)	
Pacific	18 (26.47%)	61 (17.58%)	79 (19.04%)	
Hospital bed-size				P = 0.864
Small	10 (13.24%)	49 (14.12%)	59 (13.98%)	
Medium	10 (14.71%)	44 (12.68%)	54 (13.01%)	
Large	48 (72.06%)	254 (73.20%)	302 (73.01%)	

Table 2 compares mortality, length of stay, and total hospital charges in CAR-T hospitalizations with and without CRS. The CRS group had a mortality rate of 5.89% (4/68) compared to 2.9% (10/347) in the non-CRS group. The adjusted odds ratio (aOR) for mortality was 2.48 (95% CI: 0.71 to 8.69, p = 0.151). The mean length of stay for CRS was 16.9 days, and for non-CRS, it was 18.9 days, with a coefficient of -2.1 days (95% CI: -5.43 to 1.21, p = 0.207). Mean total hospital charges were $1,148,539 for CRS and $967,146 for non-CRS, with a coefficient of $207,456 (95% CI: $6119 to $421,031, p = 0.057).

**Table 2:** Mortality, length of stay, and total hospital charges CRS: cytokine release syndrome.

	With CRS	Without CRS	Adjusted odds ratio (aOR)/coefficient (multivariable analysis)	P-value
Mortality	5.89% (4/68)	2.9% (10/347)	aOR= 2.48 (95 CI: 0.71 to 8.69)	P = 0.151
Mean length of stay	16.9 days	18.9 days	Coefficient = -2.1 days (95 CI: -5.43 days to 1.21 days)	P = 0.207
Mean hospital charges	$1,148,539	$967,146	Coefficient = $207,456 (95 CI: $6119 to $421,031)	P = 0.057

Table 3 displays the risks and outcomes associated with CRS, comparing occurrences with and without CRS. Fever was observed in 66.2% of the group with CRS and 51.9% of the group without CRS with an aOR of 1.91 (95% CI: 1.15 to 3.17, p = 0.014). Acute respiratory failure was observed in 10% of the group with CRS and 5% of the group without CRS with an aOR of 2.10 (95% CI: 1.01 to 4.40, p = 0.049). Intubation/mechanical ventilation was observed in 11.7% of the group with CRS and 5% of the group without CRS with an aOR of 2.59 (95% CI: 1.14 to 5.88, p = 0.024).

**Table 3:** Risks and outcomes associated with CRS Statistically significant p-values are in bold. CRS: cytokine release syndrome; AKI: acute kidney injury; CVA: cerebrovascular accident; MI: myocardial infarction; CHF: congestive heart failure.

Risks and outcomes	With CRS	Without CRS	Adjusted odds ratio (aOR) (multivariable analysis)	P-value
Fever	66.2% (45/68)	51.9% (180/347)	aOR = 1.91 (95 CI:1.15 to 3.17)	P = 0.014
Sepsis	8.8% (6/68)	6.9% (24/347)	aOR = 1.68 (95 CI: 0.56 to 5.04)	P = 0.348
Vasopressor support	3% (2/68)	1.4% (5/347)	aOR = 2.39 (95% CI: 0.40 to 14.35)	P = 0.334
Encephalopathy	20.6% (14/68)	22.2% (77/347)	aOR = 1.03 (95% CI: 0.50 to 2.09)	P = 0.940
Seizures	4.4% (3/68)	1.1% (4/347)	aOR = 3.96 (95% CI: 0.84 to 18.72)	P = 0.082
Acute respiratory failure	10% (7/68)	5% (17/347)	aOR = 2.10 (95% CI: 1.01 to 4.40)	P = 0.049
Intubation/mechanical ventilation	11.7% (8/68)	5% (17/347)	aOR = 2.59(95% CI: 1.14 to 5.88)	P = 0.024
*Clostridioides difficile* infection	4.4% (3/68)	5.2% (18/347)	aOR = 0.94 (95% CI:0.26 to 3.40)	P = 0.921
AKI	20.6% (14/68)	14.4% (50/347)	aOR =1.69 (95% CI: 0.84 to 3.40)	P = 0.141
Acute CVA	0% (0/68)	1.44% (5/347)	aOR = N/A	N/A
Acute liver injury	1.5% (1/68)	0.9% (3/347)	aOR = 1.71(95% CI: 0.16 to 18.24)	P = 0.651
Hepato/splenomegaly	4.4% (3/68)	2.6% (9/347)	aOR = 1.73(95% CI: 0.57 to 5.27)	P = 0.326
Acute MI	0% (0/68)	1.2% (4/347)	aOR = N/A	N/A
CHF	3% (2/68)	3.5% (12/347)	aOR = 0.85 (95% CI: 0.17 to 4.13)	P = 0.833

Other outcomes, including sepsis, vasopressor support, encephalopathy, seizures, Clostridioides difficile infection, acute kidney injury (AKI), acute cerebrovascular accident (CVA), acute liver injury, hepato/splenomegaly, acute myocardial infarction (MI), and congestive heart failure (CHF), are presented with corresponding aORs and p-values.

Table 4 outlines hematological outcomes and interventions associated with CRS. In the CRS group, hemophagocytic lymphohistiocytosis (HLH) was observed in 7.4% compared to 1.2% in the non-CRS group, with an aOR of 6.72 (95% CI: 2.03 to 22.18, p = 0.002). The risk of anemia was lower in the group with CRS compared to the group without CRS, with an aOR of 0.57 (95% CI: 0.34 to 0.94, p = 0.029).

**Table 4:** Hematological outcomes and interventions associated with CRS Statistically significant p-values are in bold. CRS: cytokine release syndrome.

Outcomes & interventions	With CRS	Without CRS	Adjusted odds ratio (aOR) (multivariable analysis)	P-value
Anemia	73.5% (50/68)	82% (284/347)	aOR = 0.57 (95% CI: 0.34 to 0.94)	P = 0.029
Thrombocytopenia	19.1% (13/68)	15% (52/347)	aOR = 1.42 (95% CI: 0.67 to 2.99)	P = 0.350
Pancytopenia	66.2% (45/68)	62.2% (216/347)	aOR = 1.40 (95% CI: 0.73 to 2.66)	P = 0.307
Hemophagocytic lymphohistiocytosis (HLH)	7.4% (5/68)	1.2% (4/347)	aOR = 6.72 (95% CI: 2.03 to 22.18)	P = 0.002
Pulmonary embolism	0% (0/68)	0.8% (3/347)	aOR = N/A	N/A
Major bleeding	0% (0/68)	2.3% (8/347)	aOR = N/A	N/A
Intracranial hemorrhage	0% (0/68)	1.4% (5/347)	aOR = N/A	N/A
Gastrointestinal bleeding	1.5% (1/68)	2.3% (8/347)	aOR = 0.67 (95% CI: 0.09 to 5.34)	P = 0.704
Disseminated intravascular coagulation	4.4% (3/68)	2.6% (9/347)	aOR = 1.73 (95% CI: 0.41 to 7.34)	P = 0.448
Red blood cell transfusion	5.8% (4/68)	9.2% (32/347)	aOR = 0.62 (95% CI: 0.23 to 1.62)	P = 0.318
Platelets transfusion	4.4% (3/68)	5.4% (19/347)	aOR = 0.89 (95% CI: 0.22 to 3.62)	P = 0.873
Fresh frozen plasma transfusion	1.5% (1/68)	0.3% (1/347)	aOR = 5.1 (95% CI: 0.30 to 90.06)	P = 0.255
Cryoprecipitate transfusion	2.9% (2/68)	2% (7/347)	aOR = 1.64 (95% CI: 0.26 to 10.34)	P = 0.590

Other outcomes and interventions, including thrombocytopenia, pancytopenia, pulmonary embolism, major bleeding, intracranial hemorrhage, gastrointestinal bleeding, disseminated intravascular coagulation (DIC), red blood cell transfusion, platelets transfusion, fresh frozen plasma (FFP) transfusion, and cryoprecipitate transfusion, are presented with corresponding aORs and p-values.

## Discussion

CAR-T therapy remains limited by significant toxicities, including CRS and ICANS. CRS occurs in around 70% of patients after CD19 CAR-T cell therapy, with published incidence rates ranging from 35% to 93%, depending on the product infused and disease treated [17]. CRS initially manifests with fever and can progress to life-threatening capillary leak with hypoxia and hypotension, with subsequent multiple organ toxicities and hematological complications. In addition to fever, cytopenias, and hypofibrinogenemia, a profound rise in the serum ferritin, soluble CD25, and cytokines, such as interferon-gamma (IFN-γ), IL2, and IL6, in severe CRS suggests many similarities to macrophage activation syndrome/hemophagocytic lymphohistiocytosis (MAS/HLH) [18,19]. The Lee scale and ASCTC (American Society for Transplantation and Cellular Therapy) grading systems categorize CRS severity into four grades: Grade 1, characterized by mild symptoms and no need for intervention; Grade 2, requiring symptomatic treatment or infusion interruption but responding promptly; Grade 3, exhibiting prolonged symptoms, recurrence after initial improvement, or hospitalization due to clinical sequelae; and Grade 4, the most severe, necessitating pressor or ventilatory support due to life-threatening consequences [20].

The present study was conducted to analyze real-world data to find risks, predictors, and outcomes of CRS in hospitalized patients treated with CAR-T therapy using the NIS. Several literature reviews and incidence studies have been conducted in the past to better understand the pathophysiology and treatment options of CRS secondary to CAR-T therapy [21,22]. However, hardly any studies have been able to conclude how the development of CRS in hospitalized patients treated with CAR-T therapy affects mortality, length of stay (LOS), demographic associations, and outcomes like organ toxicities and hematological complications [23].

Our study focused on 415 hospitalizations involving CAR-T therapy. Among these, 68 (16.38%) experienced CRS. We examined the baseline characteristics of these hospitalizations and determined that the median age was 60 years, with no significant age difference between the two groups (p-value = 0.517). The study comprised 290 males (60.24%) and 193 females (39.76%) encounters; once again, we observed no significant difference in the gender distribution for CRS incidence (p-value = 0.506). This finding aligns with other studies [24]. The study encompassed diverse racial and ethnic groups (white, black, Hispanic, Asian, Native American), and CRS incidence exhibited no significant variation, indicating no racial disparity. No significant associations were identified between CRS and the patient’s median household income percentile, as well as insurance status, including Medicare, Medicaid, private insurance, or lack of insurance. However, our study did reveal a significant association in the CRS group, with a Charlson Comorbidity Index score of 2 or more demonstrating a higher incidence of CRS. There was no significant association between CRS and different geographical locations or hospital bed sizes.

Although the incidence of mortality in the CRS group was higher in the present study sample (5.89% vs. 2.89%), there was no statistically significant difference in mortality between both study groups (aOR = 2.48; p = 0.151). This could potentially be secondary to treatment and interventions, including therapy with steroids, tocilizumab, or anakinra, along with supportive measures [25]. The difference in LOS and mean total hospital charges between both groups was found to be statistically insignificant (coefficient = -2.1 days and p = 0.207; coefficient = $207,456 and p = 0.057).

Based on our analysis, we found that the group with CRS had a significant association with fever (aOR = 1.91; p = 0.015), acute respiratory failure (aOR = 2.10; p = 0.049), and requiring intubation/mechanical ventilation (aOR = 2.59; p = 0.024). Other risks and outcomes that were studied and which showed no statistical significance were sepsis (aOR = 1.68; p = 0.348), vasopressor support requirement (aOR = 2.39; p = 0.334), encephalopathy (aOR = 1.03; p = 0.940), *Clostridioides difficile* infection (aOR = 0.94; p = 0.921), seizures (aOR = 3.96; p = 0.082), AKI (aOR = 1.69; p = 0.141), acute cerebrovascular event (p = NA), acute liver injury (aOR = 1.71; p = 0.651), hepatomegaly & splenomegaly (aOR = 1.73; p = 0.326), acute MI (p = NA), and CHF (aOR = 0.85; p = 0.833).

This study was extended to include hematological complications associated with CRS and CAR-T therapy. It revealed that the group with CRS had a statistically significant association with the development of HLH (aOR = 6.72; p = 0.002). This finding is related to the underlying pathophysiology of hyperinflammation and increased cytokine activity in CRS [18-20]. There was a statistically lower incidence of anemia in CRS-related CAR-T therapy when odds ratios were compared between the study groups (aOR = 0.5; p = 0.029), while there was a higher incidence of thrombocytopenia (19.1% vs. 15%) and pancytopenia (66.2% vs. 62.2%) in the CRS group. However, there was no statistical difference when comparing odds ratios (aOR = 1.42 and p = 0.350 for thrombocytopenia; aOR = 1.40 and p = 0.307 for pancytopenia). There were not enough reports on catastrophic bleeding, pulmonary embolism, and intracranial hemorrhage. CAR-T therapy with CRS was associated with DIC (4.4% vs. 2.6%) compared to those without CRS; however, this association was found to be insignificant (aOR = 1.73; p = 0.445). The need for red blood cell transfusion, platelet transfusion, FFP transfusion, and cryoprecipitate transfusion showed no statistically significant difference between hospitalizations of CAR-T patients with CRS and those without CRS (all p > 0.05).

Limitations

The NIS database lacks data regarding pre-admission and post-discharge information, limiting our ability to conduct long-term follow-up. Additionally, our analysis captures hospitalizations rather than individual patients, potentially resulting in duplicated data for readmissions. Furthermore, our dataset lacks significant details such as imaging, laboratory values, coagulation panel results, treatment strategies, and cause of death analyses. It is also important to note that our findings establish associations rather than causal relationships with the events we studied. Given that our data are cross-sectional in nature, it is important to interpret our findings with caution. Our analyses were carried out using retrospective registry data, which introduces the possibility of selection bias due to potential selective reporting and the use of ICD codes to define the patient cohort.

Despite these limitations, our study offers valuable insights into the occurrence of CRS in patients receiving CAR-T therapy. Our ultimate aim is to enhance patient outcomes by providing guidance for clinical decision-making. While acknowledging the existence of coding errors and variations, it is worth noting that our study is based on a substantial sample drawn from this database and represents a diverse population across the United States, and incorporates data from numerous medical centers.

## Conclusions

Based on the above analysis, it appears that the association of CRS in patients treated with CAR-T therapy did not increase the risk of mortality as well as the LOS in US hospitalized patients. The study does highlight a statistically significant association of CRS with the incidence of fever, acute respiratory failure, requirement of intubation, and HLH in patients treated with CAR-T requiring hospitalization. Although the study concludes limited risk of mortality with the development of CRS in CAR-T-treated hospitalized patients, clinicians should be vigilant with prompt recognition of CRS in such patients and initiate appropriate management.

## Appendices

**Table 5:** Supplemental table CAR-T: chimeric antigen receptor T-cell; CRS: cytokine release syndrome; AKI: acute kidney injury; CVA: cerebrovascular accident; CHF: congestive heart failure; HLH: hemophagocytic lymphohistiocytosis; DIC: disseminated intravascular coagulation; FFP: fresh frozen plasma.

Variable	ICD-10/Procedure codes used
CAR-T therapy	XW033C3, XW043C3
CRS	D89.83, D89.831, D89.832, D89.833, D89.834, D89.835, D89.839
Sepsis	A021, A227, A267, A327, A400, A401, A403, A408, A409, A41,A4101, A4102, A411, A412, A413, A414, A4150, A4151, A4152, A4153, A4159, A4181, A4189, A419, A427, A5486, B377, P360, P3610, P3619, P362, P3630, P3639, P364, P365, P368, P369, R6520, R6521, T8144XA, T8144XD, T8144XS
Vasopressor support	3E030XZ, 3E033XZ, 3E040XZ, 3E043XZ, 3E050XZ, 3E053XZ, 3E060XZ, 3E063XZ
Acute respiratory failure	J9600, J9601, J9602, J9620, J9621, J9622, J9690, J9691, J9692
Intubation/mechanical ventilation	5A09357, 5A09457, 5A09557, 09HN7BZ, 0CHY7BZ, 0DH57BZ, 0NH17EZ, 5A1935Z, 5A1945Z, 5A1955Z, 0BH07DZ
*Clostridium difficile* infection	A047, A0471, A0472
AKI	N170, N171, N172, N178, N179
Acute CVA	I639, I638, I6359, I63549, I63219, I63212, I63211, I6320, I6309, I63039, I63032, I63031, I6302, I63019, I63012, I63011, I6300
Encephalopathy	G92, G93.4, G93.40, G93.41, G93.49
Seizures	G40
Acute liver injury	K7200, K7201, K7290, K7291
Hepato &/splenomegaly	R160, R161, R162
Acute myocardial infarction	I2101, I2102, I2109, I2111, I2119, I2121, I2129, I213, I214
CHF	I50, I97. 13
Anemia	D50, D51, D52, D53, D55, D56, D57, D58, D59, D60, D61, D62, D63, D64, D46.0, D46.1, D46.2, D46.4, O99.0
Thrombocytopenia	D6942, D6949, D6959, D696
Pancytopenia	D61.81, D61.810, D61.811, D61.818
HLH	D76.1
Pulmonary embolism	I26, I260, I2602, I2609, I269, I2692, I2699
Major bleeding	R58, L7622, K661, I62, I620, I6200, I6201, I6202, I6203, I621, I629, R04, R040, R041, R042, R048, R0481, R0489, R049
Intracranial hemorrhage	I6000, I6001, I6002, I6010, I6011, I6012, I602, I6030, I6031, I6032, I604, I6050, I6051, I6052, I606, I607, I608, I609, I6030, I6030, I610, I611, I612, I613, I614, I615, I616, I618, I619, I6200, I6201, I6202, I6203, I621, I629
GI bleeding	K920, K921, K922, K625, K928, K929, K9281, K9282, K9289, K250, K254, K260, K264, K270, K28, K621
DIC	D65
Red blood cell transfusion	30233N0, 30233N1, 30243N0, 30243N1, 30273N1, 30277N1, 30233P0, 30233P1, 30243P0, 30243P1
Platelets transfusion	30233R1, 30243R0, 30243R1, 30273R1, 30277R1
FFP transfusion	30233L0, 30233L1, 30243L0, 30243L1, 30273L1, 30233K0, 30233K1, 30243K0, 30243K1, 30273K1, 30277L1, 30277K1
Cryoprecipitate transfusion	30233M0, 30233M1, 30243M0, 30243M1, 30273M1, 30277M1, 30233D1, 30243D1
